# LINC00461, a long non-coding RNA, is important for the proliferation and migration of glioma cells

**DOI:** 10.18632/oncotarget.20340

**Published:** 2017-08-18

**Authors:** Yali Yang, Mingxin Ren, Chao Song, Dan Li, Shahid Hussain Soomro, Yajie Xiong, Hongfeng Zhang, Hui Fu

**Affiliations:** ^1^ Department of Anatomy and Embryology, School of Basic Medical Sciences, Wuhan University, Wuhan 430071, China; ^2^ Department of Pathology, The Central Hospital of Wuhan, Tongji Medical College, Huazhong University of Science and Technology, Wuhan 430014, China

**Keywords:** lncRNAs, LINC00461, glioma, proliferation, migration

## Abstract

An increasing number of reports have revealed that long non-coding RNAs are important players in tumorigenesis. Here we showed that long non-coding RNA LINC00461 is highly expressed in glioma tissues compared to non-neoplastic brain tissues. The knockdown of LINC00461 suppressed cyclinD1/A/E expression which led to G0/G1 cell cycle arrest and inhibited cell proliferation in glioma cells. LINC00461 suppression also inhibited glioma cell migration and invasion. The function of LINC00461 in glioma cells is partially mediated by MAPK/ERK and PI3K/AKT signaling pathways as down-regulation of LINC00461 expression suppressed ERK1/2 and AKT activities. Moreover, LINC00461 knockdown decreased expression levels of microRNA miR-9 and flanking genes *MEF2C* and *TMEM161B.* Taken together, our results demonstrate that LINC00461 is important for glioma progression affecting cell proliferation, migration and invasion via MAPK/ERK, PI3K/AKT, and possibly other signaling pathways.

## INTRODUCTION

Long non-coding RNAs (lncRNAs) are defined as transcripts of greater than 200 nucleotides without evident protein coding function [1, 2]. In the whole genome, the protein coding portion constitutes approximately 1.5% [3], while 70%-90% portion is transcribed to produce a large transcriptome of lncRNAs [4]. LncRNAs are involved in a variety of biological functions such as chromosomal dosage compensation, imprinting, epigenetic regulation, apoptosis, cell cycle control, cell growth and differentiation [5–10]. Therefore, aberrant lncRNA expression can cause human cancers [11], such as breast cancer [12, 13], colorectal cancer [14, 15], hepato-cellular cancer [15–17], prostate cancer [18, 19], non-small cell lung cancer [20], glioma [21] and others [22].

Gliomas account for the majority of malignant brain tumors in adults, causing approximately 80% of all malignant brain tumors [23]. According to the Histopathology, the World Health Organization (WHO) has divided gliomas into four grades (I-IV) [24]. Gliomas are rarely curable especially for glioblastoma (grade IV) [25]. Current standard therapy for glioblastomas includes maximal safe surgical resection, followed by concurrent radiation with temozolomide (TMZ), an oral alkylating agent [26]. But the treatment is not effective due to the high proliferation rate and aggressive invasion of glioblastomas. The median survival of patients is merely 15 months [25]. Thus, it is urgent to advance our understanding of regulatory mechanisms involved in the initiation and progression of gliomas for developing novel and effective therapeutic approaches.

Here, we have reported that lncRNA LINC00461 is up-regulated in human gliomas. LINC00461 is located at an intergenic region of human chromosome 5 between two protein-coding genes *MEF2C* (myocyte enhancer factor 2C) and *TMEM161B* (transmembrane protein 161B). Our study suggested that LINC00461 is important for glioma cell proliferation, migration and invasion. Furthermore, we found that LINC00461 could potentially activate MAPK/ERK and PI3K/AKT pathways and expression levels of genes in its vicinity as well.

## RESULTS

### LINC00461 is expressed in neural stem/glioma cells

**Figure 1:**
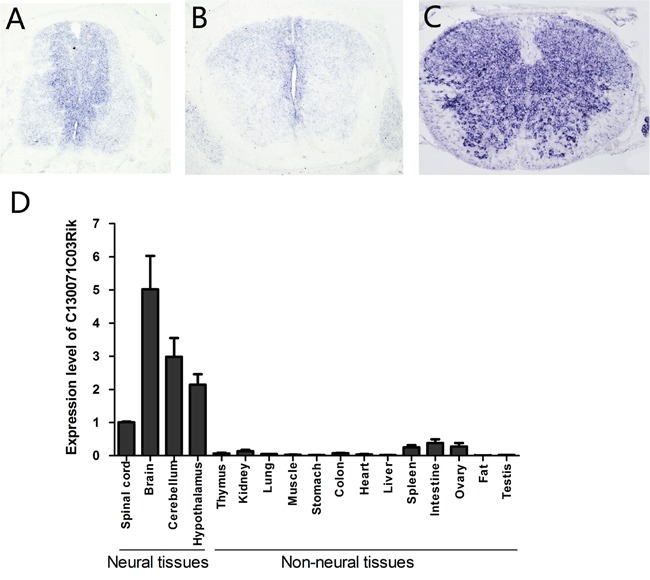
Mouse lncRNA C130071C03Rik is specifically expressed in neural stem cells during development and highly enriched in neural tissues in adults The expression of C130071C03Rik was detected in mouse spinal cord at E11.5 **(A)**, E13.5 **(B)**, and P0 **(C)** by *in situ* hybridization. **(D)** Relative expression levels of C130071C03Rik in different mouse tissues/organs were measured by real-time PCR at P60. The average expression level of C130071C03Rik in the spinal cord was set as 1. Data are presented as mean ± SEM.

Previously, we compared transcriptomes of mouse spinal cords at E13.5 (embryonic day 13.5) with those at P0 (postnatal day 0) and identified several genes that are highly expressed at E13.5, including lncRNA C130071C03Rik. Now further studies revealed that it is specifically expressed in the ventricular zone of the mouse spinal cord at E11.5 (Figure 1A) and E13.5 (Figure 1B), where neural stem/precursor cells are located. At P0, its expression spreads out to the whole spinal cord (Figure 1C). In the mouse brain, we detected its expression in the subventricular zone (SVZ) at P0 (Supplementary Figure 1A, 1B). Real-time PCR analysis showed that C130071C03Rik is highly expressed in mouse neural tissues compared to non-neural tissues (Figure 1D).

**Figure 2:**
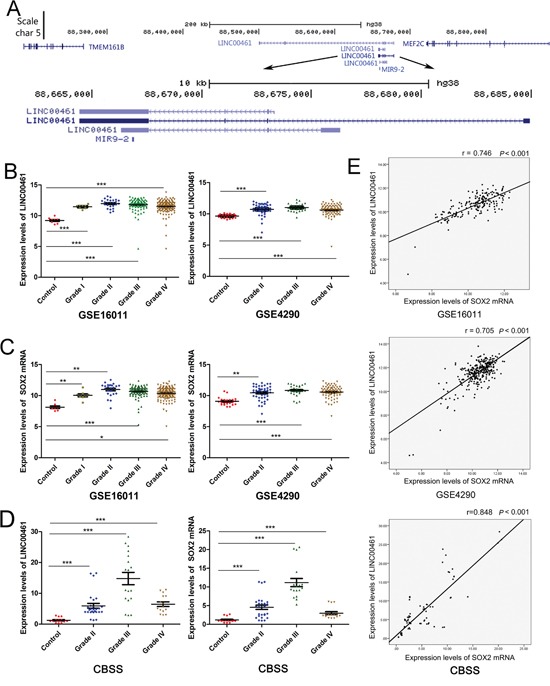
Expression levels of LINC00461 are up-regulated in glioma tissues and positively correlated with those of SOX2 **(A)** UCSC genome browser view of the LINC00461 locus in the human genome. **(B)** Expression levels of LINC00461 were analyzed in GSE16011 and GSE4290 glioma datasets. **(C)** Expression levels of SOX2 mRNA were analyzed in GSE16011 and GSE4290 glioma datasets. **(D)** Expression levels of LINC00461 and SOX2 in 5 nonneoplastic brain tissues and 19 glioma tissues were measured by real-time PCR in Chinese brain sample set (CBSS). **(E)** The expression of LINC00461 positively correlated with that of SOX2 in GSE16011, GSE4290, and CBSS. Each sample has been measured three times. Data are presented as mean ± SEM. ^*^, *P* < 0.05; ^**^, *P*< 0.01; ^***^, *P*< 0.001.

The liftOver program was used to identify single mapped orthologous regions in genomes of diverse species. We found that the ortholog of lncRNA C130071C03Rik in humans was LINC00461. LINC00461 is transcribed from an intergenic region of human chromosome 5 between *MEF2C* and *TMEM161B* (Figure 2A). Using *in situ* hybridization (ISH) technique, we demonstrated that LINC00461 transcript predominantly locates in the cytoplasm of U251 and U87MG glioma cells (Supplementary Figure 1C).

It has been reported that glioblastoma cells harbor some properties similar to those of neural stem cells. Their gene expression profiles resemble each other. Therefore we decided to find out whether this lncRNA is enriched in gliomas. Glioma datasets GSE16011 and GSE4290 showed that expression levels of LINC00461 were up-regulated in gliomas (grade I-IV in GSE16011, grade II-IV in GSE4290) compared to the control (Figure 2B). We also checked the expression of SOX2, a neural stem cell marker, and found that SOX2 mRNA expression levels were also up-regulated in gliomas (Figure 2C). In our own Chinese glioma collection, we found that expression levels of both LINC00461 and SOX2 mRNAs were markedly higher in gliomas than those in non-neoplastic brain tissues (*P* < 0.001) (Figure 2D). Pearson correlation analysis revealed significant and positive correlation between LINC00461 and SOX2 mRNAs in GSE16011 and GSE4290 datasets (Figure 2E). Again, a positive correlation between mRNA levels of LINC00461 and SOX2 was detected in Chinese glioma samples (Figure 2E). Up-regulation of SOX2 has been linked to the development and maintenance of gliomas. Our findings suggested that LINC00461 might be involved in the development of gliomas, regulating stem-cell like properties in gliomas.

### The knockdown of LINC00461 decreased cell viability of glioma cells, while had no effects on cell apoptosis

**Figure 3:**
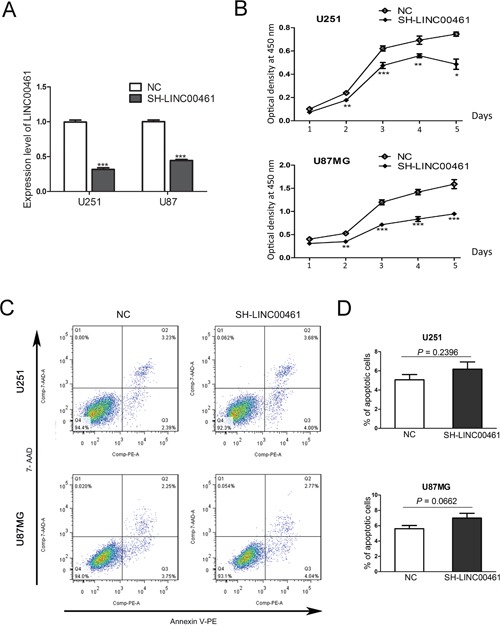
The knockdown of LINC00461 decreased cell viability with no effects on cell apoptosis **(A)** The efficiency of LINC00461 knockdown in both U251 and U87MG cells was measured by real-time PCR. **(B)** The cell viability was measured by CCK-8 assay. The optical density at 450 nm was used as the positive index of cell viability. **(C)** The cell apoptosis was analyzed by Annexin V-PE/7-AAD assay. **(D)** Histograms showing total apoptotic rates (containing early and late apoptosis rates) in U251 and U87MG cells. Cells treated with scrambled sequences were used as the negative control. SH-LINC00461: shRNA#1 against LINC00461; NC: negative control. Data are presented as mean ± SEM. ^*^, *P* < 0.05; ^**^, *P*< 0.01; ^***^, *P*< 0.001.

Lentivirus-mediated short hairpin RNAs (shRNAs) were applied to knockdown LINC00461 expression. 48 hours after lentivirus infection, expression levels of LINC00461 were measured by real-time PCR to determine the effect of LINC00461 shRNA. We had designed two different shRNAs. Both significantly suppressed expression levels of LINC00461 in U251 and U87MG cells (Figure 3A, Supplementary Figure 3A) and reduced the cell viability at 2, 3, 4 and 5 days post the lentivirus treatment (Figure 3B, Supplementary Figure 3B).

Then we decided to find out whether LINC00461 knockdown induces cell apoptosis. For this purpose, Annexin V-PE/7-AAD assays were performed to measure numbers of cells that were viable (Annexin v-, 7-AAD-), underwent early apoptosis (Annexin v+, 7-AAD-), late apoptosis (Annexin v+, 7-AAD+), or necrosis (Annexin v-,7-AAD+) (Figure 3C). Apoptosis rates (early plus late apoptosis) after LINC00461 shRNA treatment were 6.18±1.30% in U251 cells and 7.01 ± 1.07% in U87MG cells, similar to those in control groups (5.1 ± 0.56% in U251 cells and 5.61 ± 0.42% in U87MG cells) (Figure 3D). Statistical analysis showed no significant difference between treatment and control groups (*P* = 0.2396 in U251 cells and *P* = 0.0662 in U87MG cells) (Figure 3D). We also measured expression levels of apoptotic protein caspase-3 after the shRNA treatment. Results showed that protein levels of caspase-3 and cleaved caspase-3 were not affected by LINC00461 shRNA (Supplementary Figure 2). These results indicated that knockdown of LINC00461 has little effect on apoptosis.

### LINC00461 knockdown led to G0/G1 phase arrest and inhibited cell proliferation

**Figure 4:**
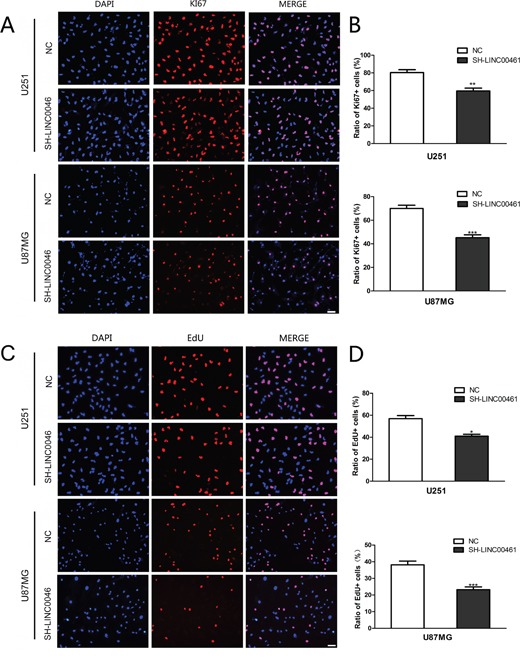
LINC00461 knockdown inhibited cell proliferation **(A)** Images of U251 and U87MG cells stained with the antibody against ki67. **(B)** Histograms showing ratios of proliferating ki67 positive cells. **(C)** Cell proliferation was measured by EdU assay in U251 and U87MG cells. **(D)** Histograms showing ratios of EdU positive cells. Data are presented as mean ± SEM. ^*^, *P* < 0.05; ^**^, *P* < 0.01; ^***^, *P* < 0.001. Scale bar: 50 μm.

To evaluate the effect of LINC00461 on cell proliferation, we used both ki67 antibody and EdU staining to measure the proliferation rate. Ki67 antibody can recognize a protein that is present during all active phases of the cell cycle, but absent in the resting stage. EdU is an analog for thymidine and can be incorporated into the newly synthesized DNA at S phase of the cell cycle. The percentage of Ki67 positive cells after LINC00461 shRNA treatment was 59.50 ± 0.33% in U251 cells and 45.20 ± 0.23% in U87MG cells, while in control groups it was 79.18 ± 0.40% in U251 cells (*P* < 0.01) and 69.95 ± 0.26% in U87MG cells (*P* < 0.001) (Figure 4A, 4B). Results were confirmed by EdU staining experiments. In LINC00461 knockdown groups, the percentage of EdU positive cells was 40.98 ± 1.68% in U251 cells and 23.23% ± 1.64% in U87MG cells, compared to 56.91 ± 2.79% (*P* < 0.05) in U251 cells and 38.15 ± 2.24% (*P* < 0.001) in U87MG cells for control groups (Figure 4C, 4D). Our results suggested that suppressed expression of LINC00461 inhibits cell proliferation which contributes to the reduction of cell viability.

**Figure 5:**
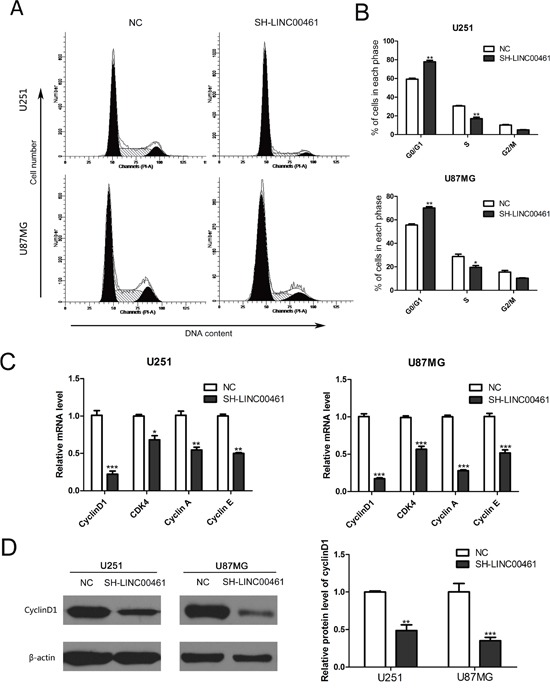
The knockdown of LINC00461 caused G0/G1 phase arrest and reduced expression of cyclins and CDK4 **(A)** After lentivirus infection, cell cycle distribution was analyzed by flow cytometry. **(B)** Histograms showing cell cycle phase distribution of both cells. **(C)** Expression levels of cyclinD1, CDK4, cyclin A and cyclin E mRNAs were measured by real-time PCR. **(D)** Expression levels of cyclinD1 protein were analyzed by the Western blotting. Histograms showed the quantization of Western blots (normalized by β-actin expression). Data are presented as mean ± SEM. ^*^, *P* < 0.05; ^**^, *P* < 0.01; ^***^, *P* < 0.001.

In order to explore mechanisms underlying the anti-proliferation effect of LINC00461 knockdown on glioma cells, we analyzed cell cycle distribution by flow cytometry. As showed in Figure 5, 48 hours after down-regulation of LINC00461, the fraction of cells in G0/G1 phase increased to 77.86 ± 1.59% in U251 cells compared to 59.31 ± 1.07% in control cells (Figure 5A, 5B). Similar results were obtained with U87MG cells. The fraction of cells in G0/G1 phase increased to 70.14 ± 1.13% in experimental groups, whereas the fraction in control group was 55.65±0.95% (Figure 5A, 5B). The fraction of cells in S phase decreased significantly after LINC00461 shRNA treatment (treatment group 17.08% ± 1.55% vs. control group 30.57 ± 0.56%in U251 cells, 19.57 ± 1.52% vs. 28.79 ±2.06% in U87MG cells) (Figure 5A, 5B).

We further measured expression levels of some proteins important for regulating the cell cycle. In LINC00461 knockdown groups, expression levels of cyclinD1, CDK4, cyclin A, cyclin E mRNAs significantly decreased in U251 and U87MG cells (Figure 5C). Moreover, expression levels of cyclinD1 protein decreased about 50% in U251 cells (*P* < 0.01) and 65% in U87MG cells (*P* < 0.001) (Figure 5D). These results showed that down-regulation of LINC00461 can reduce expression levels of cyclinD1, CDK4, cyclin A and cyclin E, which leads to G0/G1 phase arrest and the delay of cell cycle progression.

### LINC00461 knockdown inhibited cell migration and invasion

**Figure 6:**
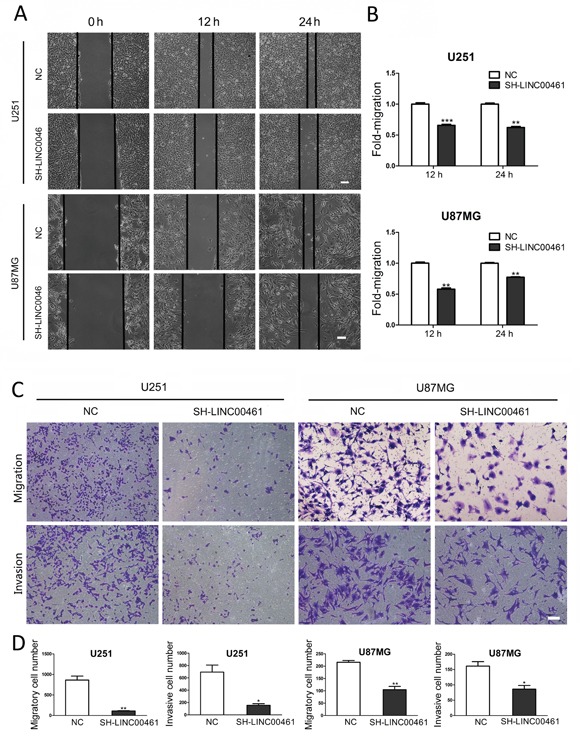
Down-regulation of LINC00461 inhibited cell migration and invasion **(A)** The wound healing assay showed delayed gap closure in LINC00461 knockdown cells compared with that in control cells at 12 h and 24 h time points in U251 and U87MG cells. **(B)** Wound gaps were analyzed by measuring the distance of migrating cells. The migrating distance by control cells is set as 1. **(C)** Representative images of Transwell assay showing the effect of LINC00461 knockdown on cell migration and invasion. **(D)** Histograms showing numbers of migratory and invasive cells. Data are presented as mean ± SEM. ^*^, *P* < 0.05; ^**^, *P* < 0.01; ^***^, *P* < 0.001. Scale bar: 100 μm.

We tried to find out whether down-regulation of LINC00461 could affect cell migration and invasion, which are key determinants for tumor aggressiveness and metastasis. Wound-healing assay was performed to investigate the effect of LINC00461 on cell migration. At the time points of 12 h and 24 h after wound scraping, slow migration in LINC00461 knockdown group was observed in both U251 and U87MG cells (Figure 6A, 6B, Supplementary Figure 3C). Transwell assay was also used to further verify the effect of LINC00461 on migratory and invasive properties of tumor cells. After LINC00461 shRNA treatment, numbers of cells underwent migration and invasion were markedly reduced in U251 (*P* < 0.01 in migration assay and *P* < 0.05 in invasion assay) and U87MG cells (*P* < 0.01 in migration assay and *P* < 0.05 in invasion assay) (Figure 6C, 6D). These data showed that knockdown of LINC00461 reduces migratory and invasive properties of glioma cells.

### LINC00461 knockdown suppressed expression levels of markers for stem cells and EMT

**Figure 7:**
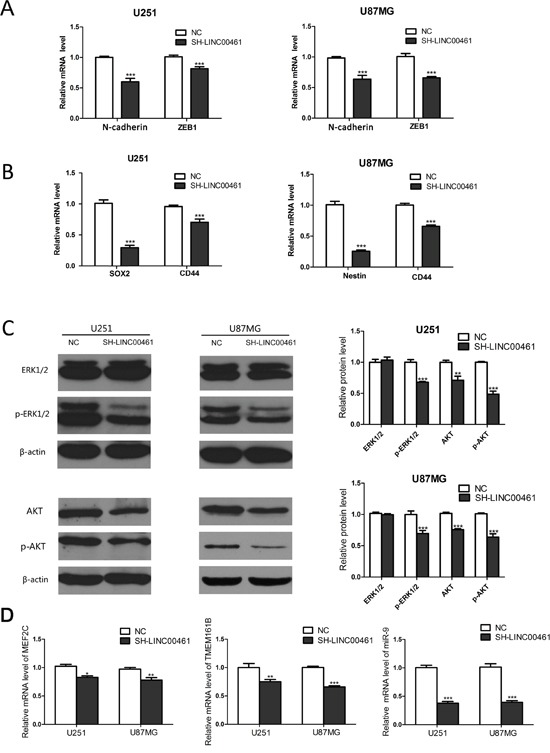
Mechanisms of LINC00461 promoting glioma cell proliferation, migration and invasion **(A)** Expression levels of N-cadherin and ZEB1 mRNA were measured by real-time PCR. **(B)** Expression levels of SOX2, CD44 and Nestin mRNA were measured by real-time PCR. **(C)** Western blots showed that down-regulation of LINC00461 suppressed AKT, p-AKT and p-ERK1/2 in U251 and U87MG cells. Histograms showed relative mean values of protein levels from three independent experiments. **(D)** Analysis on expression levels of two protein-coding and one non-coding transcripts in close proximity to the LINC00461 locus. Data are presented as mean ± SEM. ^*^, *P* <0.05; ^**^, *P* <0.01; ^***^, *P* <0.001.

Our data have showed that LINC00461 is highly expressed in neural stem cells and increases cell proliferation and migration, which are important features of stem cells. We decided to find out whether LINC00461 regulates “stemness” of glioma cells. We chose some classic stem cell markers, such as SOX2, Nestin and CD44. The result showed that LINC00461 knockdown significantly reduced mRNA levels of stem cell markers: SOX2 and CD44 in U251 cells, Nestin and CD44 in U87MG cells (Figure 7C).

It is well known that cancer cells usually undergo EMT to gain migratory and invasive properties. So we analyzed expression levels of EMT markers as well. Real-time PCR results showed that LINC00461 knockdown suppressed mRNA levels of EMT markers, such as N-cadherin and ZEB1 (Figure 7B), in U251 and U87MG cells.

### Potential mechanisms for LINC00461 promoting glioma cell proliferation, migration and invasion

MAPK/ERK pathway is well known for its roles in oncogenesis. Phosphatidylinositol 3-kinase (PI3K)/AKT signaling pathway also plays a major role in cell proliferation, migration and invasion, especially in glioma cells. In order to find out the mechanism underlying the regulation of LINC00461 on glioma cells, we studied effects of LINC00461 on different signaling pathways. Expression levels of total and phosphorylated AKT and ERK1/2 proteins were measured in glioma cells by Western blot technique. Results showed that LINC00461 knockdown significantly suppressed phosphorylation of ERK1/2 proteins (*P* < 0.001) in both U251 and U87MG cells (Figure 7A, 7B). Moreover, down-regulation of LINC00461 also decreased expression levels of total AKT (*P* < 0.01) and phosphorylated AKT proteins (*P* < 0.001) in both U251 and U87MG cells (Figure 7A, 7B). These results indicated that LINC00461 might promote glioma cell proliferation, migration, and invasion through MAPK/ERK and PI3K/AKT signaling pathways.

Studies have shown that lncRNAs can regulate genes in its vicinity via *cis*-interaction. There are two protein-coding genes *MEF2C* and *TMEM161B* at either side of LINC00461. LINC00461 itself also contains a microRNA miRNA-9-2, which can produce a mature microRNA miR-9. In our study, after the knockdown of LINC00461, MEF2C mRNA levels were reduced to 82.80 ± 2.66% in U251 (*P* < 0.05) and 77.88 ± 4.47% in U87MG cells (*P* < 0.01) (Figure 7C). Expression levels of TMEM161B mRNA also decreased to 75.86 ± 3.82% in U251 cells (*P* < 0.01) and 65.28 ± 2.23% in U87MG cells (*P* < 0.001) (Figure 7C). The knockdown of LINC00461 also drastically suppressed expression levels of miR-9 to 37.71 ± 3.08% in U251 (*P* < 0.001) and 39.26 ±2.92% in U87MG cells (*P* < 0.001) (Figure 7C). Our data showed that LINC00461 can affect nearby genes possibly via cis-interaction.

## DISCUSSION

We have identified a mouse lncRNA C130071C03Rik which is highly expressed in mouse neural tissues. Our studies showed that it is specifically expressed in mouse neural stem cells during development. Its human homolog, LINC00461, is up-regulated in human glioma tissues. We performed a series of experiments and found that LINC00461 plays multiple roles in glioma cells, affecting cell proliferation, migration, and invasion. Our results also suggested that it has little effect on cell apoptosis. The knockdown of LINC00461 caused cells being arrested during G1/S transition with concomitant decreased cyclinD1/A/E expression. We also found that LINC00461 is involved in MAPK/ERK and PI3K/AKT signaling pathways and regulates genes in its vicinity.

There are very few studies related to this lncRNA. It has been reported by Oliver et al. in 2015. They found that *Visc-1* (*aka*. C130071C03Rik) was expressed in the cortical plate and the ventricular and subventricular zones of the telencephalon as well as the rostral and caudal interneuron migratory streams at E16.5 [27]. Their data suggested its role in cell proliferation and migration, which is consistent with our findings. LINC00461 has also been reported to be up-regulated in several types of cancers. For example, Pastori et al. have reported LINC00461 as one of the top 100 most up-regulated lncRNAs in glioblastomas [28]. Crea et al. also showed that LINC00461 is up-regulated in metastatic prostate cancers [29].

In our study, we found that mouse ortholog of LINC00461 is expressed in the ventricular zone of mouse spinal cord at E11.5 and E13.5. It is also highly enriched in gliomas. Further studies revealed significant and positive correlation between LINC00461 and SOX2 mRNA in gliomas. SOX2 activity has been found to be associated with the maintenance of the undifferentiated state of cancer stem cells [30]. These results suggested that LINC00461 may regulate “stem-like” properties of neural/cancer stem cells.

LINC00461 plays multiple functional roles in glioma cells. One of them is cell cycle regulation. The loss of the regulatory control of the cell cycle, which leads to an unrestrained cell proliferation, is a hallmark of cancer cells [31]. Our data suggested that LINC00461 knockdown could induce the G1/S transition arrest which leads to the decreased viability of glioma cells. Further, we found that suppression of LINC00461 resulted in decreased expression levels of cyclinD1/E/A and CDK4. CyclinD1 plays pivotal roles in G1/S transition and is frequently over-expressed in a large number of cancers including gliomas. Its high expression is associated with the tumor malignancy and poor prognosis. CyclinD1 binds to CDK4 and CDK6 (cyclin-dependent kinase 4/6) to phosphorylate retinoblastoma protein and activate the transcription factor E2F-1. E2F-1 initiates the transcription of some cell cycle regulators, such as cyclin E and cyclin A, to drive the transition from G1 to S phase [32].

In most epithelial carcinomas, cancer cells usually undergo the epithelial-mesenchymal transition (EMT) to gain invasiveness and start metastasis. During the EMT process, several EMT markers or inducers, including ZEB1 and SNAIL, are activated to promote the “stemness” of cancer cells, thus induce cancer invasion [33]. In our study, we found that reduced LINC00461 expression reduced expression levels of some EMT markers (N-cadherin and ZEB1) and several other factors related to glioma “stemness” (CD44, SOX2, Nestin). Therefore, LINC00461 may participate in glioma progression via promoting EMT.

Current studies have found lncRNAs play critical roles in the progression of gliomas. HOTAIR (HOX transcript antisense intergenic RNA) has been reported to be up-regulated in gliomas. Its knockdown inhibits cell proliferation, migration, invasion, promotes apoptosis and cell cycle arrest by up-regulating miR-326 in human glioma cells [34]. Wang et al. have reported that lncRNA CRNDE (colorectal neoplasia differentially expressed) is up-regulated in gliomas and promotes glioma cell growth and invasion through mTOR signaling pathway [21]. Shi et al. have found that lncRNA H19 is closely correlated with the severity of tumors. H19 transcript can produce a miRNA (miR-675), which directly regulates CDH13 (Cadherin 13), thereby modulating glioma cell invasion [35].

In this study, we have found that LINC00461 regulates miRNA miR-9, which is the mature form of miR-9-2 gene. The sequence of miR-9-2 is included in the sequence of LINC00461. Knockdown of LINC00461 significantly suppressed expression levels of miR-9. Wu et al. have reported that expression levels of miR-9 in gliomas are significantly higher than those in non-neoplastic brain tissues [36]. The overall survival time of glioma patients with high miR-9 expression levels is obviously shorter than that with low miR-9 expression levels [36]. Schraivogel et al. found that miR-9 promotes neurosphere-like formation of glioblastoma stem cells by targeting a tumor suppressor CAMTA1 (calmodulin-binding transcription activator 1) [37]. It has also been reported that miR-9 can promote glioma cell migration by repression of NF1 (neurofibromin1) [38]. Based on these studies and our own work, we concluded that LINC00461 might regulate glioma progression via miR-9.

Studies have shown that miR-9 could target multiple signaling pathways [39]. In ovarian cancer and gastric cancer, miR-9 has been shown to target NF-κB pathway leading to inhibition of cell proliferation and metastasis [40, 41]. On the other hand, miR-9 overexpression has been shown to enhance metastasis in esophageal squamous cell carcinoma by targeting E-cadherin [42]. Zhang et.al have found that overexpression miR-9 could block the phosphorylation of AKT (Ser473) and ERK [42]. In our study, we found that overexpression or inhibitor of miR-9 has no effects on AKT and ERK in glioma cells (Supplementary Figure 4). Further studies are needed to decipher the mechanism of miR-9 regulation on gliomas.

Recent studies have found that long intergenic non-coding RNAs (lincRNAs) can regulate expression levels of protein-coding genes in their vicinity [43]. We found that LINC00461 is important for the expression of neighbor genes, *TMEM161B* and *MEF2C*, possibly via *cis-*interaction. Expression levels of both genes decreased after the treatment of LINC00461 shRNA. The function of TMEM161B is still not clear, while MEF2C has been reported to play an anti-apoptotic role, protecting the differentiating cells from death during neurogenesis [44], and promote neuronal synapse formation [45, 46]. Li et al. showed that *Mef2c* knockout in Nestin-expressing neural stem/progenitor cells (NSCs) impaired neuronal differentiation *in vivo*, resulting in aberrant cell compaction and smaller somal size. However, the proliferation and survival of NSCs were not affected [47]. Still no reports have been published about effects of MEF2C on gliomas.

**Figure 8:**
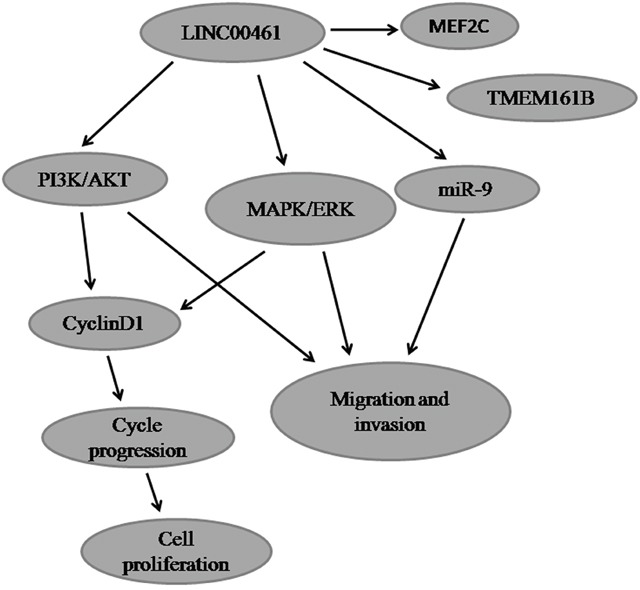
The cartoon of the mechanism underlying the regulation of LINC00461 on glioma cells

Previous studies have shown that several signaling pathways are involved in gliomas [48]. Among them, MAPK/ERK and PI3K/AKT are two frequently activated pathways [49, 50]. ERK1/2 (Extracellular signal-regulated kinases 1 and 2) belong to the mitogen-activated protein kinase (MAPK) family and play crucial roles in a wide range of biological processes, including cell proliferation, differentiation, and motility. They have been reported to be activated in glioblastomas [51]. Meanwhile, elevated AKT phosphorylation levels have been observed in 84% cases of glioblastomas [52]. The PI3K/AKT signaling pathway plays fundamental roles in regulating cellular processes such as cell proliferation, survival, and migration [53]. Our studies found that LINC00461 positively affects both MAPK/ERK and PI3K/AKT pathways. Knockdown of LINC00461 can suppress not only phosphorylation levels of ERK1/2 and AKT proteins, but also total AKT protein levels. Dual activation of MAPK/ERK and PI3K/AKT signaling pathways has been reported in many types of cancers. In thyroid cancers, both MAPK/ERK and PI3K/AKT pathways are reported to be activated, which play fundamental roles in promoting cell growth, survival and are associated with tumorigenesis [54, 55]. Lv et al. found that CXCR4 knockdown repressed glioma cell migration via deactivation of both ERK and PI3K/AKT pathways [56]. Our laboratory previously has found that TCF3 (transcription factor 3) can activate both MAPK/ERK and PI3K/AKT signaling pathways to suppress cell apoptosis and promote cell migration [57]. These data suggested that LINC00461 might promote glioma cell proliferation, migration, and invasion via both MAPK/ERK and PI3K/AKT signaling pathways (Figure 8).

It is worthwhile to note that cyclinD1 can be regulated by a number of signaling pathways, including PI3K/AKT and ERK1/2 pathways. PI3K/AKT pathway can promote cyclinD1 expression and positively regulates G1/S cell cycle progression through deactivation of GSK3-bata [58]. In addition, persistent activation of ERK1/2 is required to pass the G1 restriction point in fibroblasts [59, 60]. Our data suggested that LINC00461 can activate both MAPK/ERK and PI3K/AKT signaling pathways which lead to cyclinD1 activation and cell proliferation (Figure 8).

In summary, our studies suggested that lncRNA LINC00461 plays important roles in gliomas via recruiting multiple pathways. Based on these studies, LINC00461 could be a potential target in glioma diagnosis and treatment.

## MATERIALS AND METHODS

### Human tissues

Glioma gene expression data were downloaded from the Gene Expression Omnibus Web site (http://www.ncbi.nlm.gov/geo/, GSE16011 and GSE4290). GSE16011 dataset contains 276 glioma samples (8 samples of grade I, 24 samples of grade II, 85 samples of grade III and 159 samples of grade IV) and 8 samples of nonneoplastic brain tissues. GSE4290 dataset contains 157 glioma samples (45 samples of grade II, 31 samples of grade III, and 81 samples of grade IV) and 23 samples of nonneoplastic brain tissues. Chinese gliomas and nonneoplastic brain tissues were collected from the Department of Neurosurgery, Renmin Hospital of Wuhan University from year 2013 to 2015. Informed consent was obtained from each patient. The study was approved by the Institutional Research Board of Renmin Hospital. No prior treatments had been given to these patients. Samples were examined and verified by pathologists. All specimens were immediately frozen in liquid nitrogen after resection and kept in liquid nitrogen for storage. Chinese glioma specimens included 9 samples of grade II, 5 samples of grade III and 5 samples of grade IV. Five cases of nonneoplastic brain samples were collected from patients with cerebral trauma.

### Cell culture

U251 and U87MG human glioma cells were maintained in alpha-modification of eagle's medium (α-MEM) or Dulbecco's Modified Eagle Medium (DMEM) with high glucose (Hyclone, USA) and supplemented with 10% fetal bovine serum (Sigma, USA). Cells were kept in an incubator at 37°C, a minimum relative humidity of 95%, and atmosphere of 5% CO_2_ in air.

### RNA extraction, reverse-transcription and real-time PCR

Total RNA from tissues or cells was extracted with Trizol reagent (Invitrogen, USA) under the manufacturer's instruction. RNA concentration was measured by NanoDrop-2000 (Thermo Fisher, USA). 2.5 μg of total RNA was used for cDNA synthesis with RevertAid First Strand cDNA Synthesis kit (Thermo Fisher, USA). Expression levels of mature miR-9 were detected by the Bulge-LoopTM miRNA q-RT-PCR primer kits (RiboBio, Guangzhou, China). Roche light Cycler 480 mix (Roche, Mannheim, Germany) was employed to perform real-time PCR on a CFX 96 Real-Time PCR Detection System (Bio-rad, USA). GAPDH mRNA or U6 snRNA was used as the internal control. Sequences of real-time PCR primers were listed in Table 1.

**Table 1:** Sequences of real-time PCR primers

	Forward (5' to 3')	Reverse (5' to 3')
AP3B1	GAAGCGGATTGTTGGGATGAT	TCAGCATATCGAACCAGGTAAAC
C130071C03Rik	CATTTCCCACCCACAGCCATCT	CTCTTGGCACCCTTTCCACTTG
CD44	CTGCCGCTTTGCAGGTGTA	CATTGTGGGCAAGGTGCTATT
CDK4	ATGGCTACCTCTCGATATGAGC	CATTGGGGACTCTCACACTCT
CDK6	CCAGATGGCTCTAACCTCAGT	AACTTCCACGAAAAAGAGGCTT
CyclinA	CGCTGGCGGTACTGAAGTC	GAGGAACGGTGACATGCTCAT
CyclinD1	TGCGAAGTGGAAACCATCCG	AGGAAGCGGTCCAGGTAGTT
CyclinE	GCCAGCCTTGGGACAATAATG	CTTGCACGTTGAGTTTGGGT
GAPDH	GGGAGCCAAAAGGGTCATCA	TGATGGCATGGACTGTGGTC
LINC00461	GACATTTACGCCACAACCCACG	AGACAGACCCTCAGATTCCCCA
MEF2C	TTCACTGTTGTGCTCCTTTGC	CGGGTCTGTCCAAGCATCTAT
N-cadherin	AGCCAACCTTAACTGAGGAGT	GGCAAGTTGATTGGAGGGATG
Nestin	CTGCTACCCTTGAGACACCTG	GGGCTCTGATCTCTGCATCTAC
ONECUT2	CAAACGCCCGTCAAAGGAGAT	GCTCAGATCGTCTTGCCACTT
SOX2	TACAGCATGTCCTACTCGCAG	GAGGAAGAGGTAACCACAGGG
TMEM161B	AAGAAAGAAGCGGGGCGAAT	GCAGAGTTGTGTGAAGCAGC
ZEB1	CAGCTTGATACCTGTGAATGGG	TATCTGTGGTCGTGTGGGACT

The 2^-Δ ΔCt^ method was applied to analyze real-time PCR results.

### In site hybridization

C57BL/6 mouse spinal cords and brains were fixed in 4% paraformaldehyde (PFA) at 4°C overnight. Targeted sequences for riboprobes were amplified by PCR and cloned into pBLUE-T plasmid (Zoman, Beijing, China). Primer sequences for riboprobe cloning were: C130071C03Rik forward 5’-CCGAAGACTTAGAGCTAGCAGG-3’, reverse 5’- TCACAAGGCCCTAACCACATAC-3’, LINC00461 forward 5’-TCTCTGTTCCAAGAGGGTTTCC-3’, reverse 5’-GCTGTTTCCTGGATAGACCTGAT-3’. Protocols for tissue preparation and *in situ* hybridization with digoxigenin-labeled probes have been previously described [61].

### Lentivirus preparation

Annealed DNA fragments containing either scrambled sequences or short-hairpin RNA (shRNA) sequences targeting LINC00461 were cloned into plasmid PLKO.1 ZSGreen (Anti-hela biotech, Xiamen, China). Then, PLKO.1 vector was co-transfected with pMD2.G and psPAX2 plasmids into HEK293T cells using Neofect DNA transfection reagent (Neofect Tech, Beijing, China). ShRNA targeting sequences were: shLINC00461 5’-GGAAATGAAAGTGACATTTAC-3’, the second shRNA shRNA#2: 5’-GGAAGCTACTGAAGCAGAAAG-3’, and scrambled sequences 5’- CCTAAGGTTAAGTCGCCCTCG-3’. The scrambled sequences were used as the negative control (NC) for shRNA knockdown experiments.

### MiRNA transfection

MiR-9 mimics, non-specific control, miR-9 inhibitor, and random sequence were all purchased from RiboBio (Guangzhou, China). Cells were transfected at 40-60% confluence using Neofect siRNA transfection reagent (Neofect Tech, Beijing, China).

### Western blotting analysis

48 h after lentivirus infection, total protein was extracted from cells by RIPA cell lysis buffer with proteinase and phosphatase inhibitors. The supernatant of lysates was collected and electrophoresed in 10%-12% SDS-PAGE gel, then transferred onto PVDF membranes. Membranes were blocked in Tris-buffered saline plus 0.1% Tween-20 (TBST) with 5% non-fat dry milk or bovine serum albumin (BSA) at room temperature for 1h, followed by primary antibody incubation at 4°C overnight. On the next day after washing with TBST, membranes were incubated with secondary antibodies labeled with HRP at room temperature for 1 h. Then proteins were detected by ECL detection system (P1010, Applygen Technology, Beijing, China). Protein levels were measured by Image J software. Primary antibodies were listed as follows: caspase-3(No. 9662, 1:1000, Cell Signaling Technology), cleaved caspase-3 (No. AF7022, 1:1000, Affinity Biosciences); ERK1/2 antibody (No.4695, 1:1000, Cell Signaling Technology); p-ERK1/2 (Thr202/Tyr204) antibody (No. 4370, 1:1000, Cell Signaling Technology); AKT (No. 9272, 1:1000, Cell Signaling Technology), p-AKT (Ser473) antibody (No. 9271,1:1000, Cell Signaling Technology); cyclinD1 (AB134175, 1:20000,Abcam); β-actin (No. 10494-1-AP, 1:10000, Proteintech, Beijing, China).

### CCK-8 assay

The CCK-8 cell counting kit (Zoman, Beijing, China) was used to determine the cell viability. U251 or U87MG cells were infected with lentivirus for 48 h. Then, cells were seeded onto 96-well plates at the density of 2 × 10^3^ cells per well for measurement at time points of 1day (d), 2d, 3d, 4d and 5d after lentivirus infection. At each time point, 10μl CCK-8 solution was added into each well. After 1 h incubation at 37°C, optical density (OD) at 450 nm was measured by a microplate reader (EXL800 from BioTek, Winooski, Vermont, USA).

### Wound healing assay

U251 and U87MG cells were seeded in 6-well plates at a density of 2 × 10^5^ cells per well. 48 h after lentivirus infection, the cell layer was wounded by a sterile 10μl pipette tip. Cells were washed three times with PBS to remove floating cells. Then cells were allowed to grow for 24 hours. The wound closure was observed by a light microscope at x100 magnification. Images of three random fields were captured at 0 h, 12 h, and 24 h time points post wounding.

### Cell migration and invasion assays

Twenty-four-well plates and Transwell chambers (pore size at 8μm) (corning, New York, USA) were used for cell migration and invasion experiments. For the migration assay, 48 h after lentivirus infection, 200 μl of serum-free medium containing 1 × 10^5^ cells was added to the upper chamber, and 600 μl of medium with 20% serum was added to the lower chamber as a chemoattractant. Cells were incubated for another 20 h at 37°C before measurement. For the invasion assay, the upper chamber was pretreated with 50 μl matrigel (BD Bioscience, San Jose, USA) for 4 h at 37°C. Then, 200 μl of serum-free medium containing 2 × 10^5^ cells was added to the upper chamber, while 600μl medium with 20% serum to the lower chamber. Cells were incubated for another 30 h at 37°C. After the incubation, cells in top chambers were removed by a cotton swab. Cells on the lower surface of the insert were fixed in 4% PFA for 15 min, and stained with 0.1% crystal violet for 30min. An optical microscope was used to visualize stained cells in five random fields.

### EdU assay

EdU assay kit (Ribobio, Guangzhou, China) was used to detect cell proliferation. 6 × 10^3^ cells were cultured for each well in 24-well plates. 48 h after lentivirus infection, 200 μl of 50 μM EdU solution was added into each well. Cells were cultured for another 2 h. Afterward, cells were fixed with 4% PFA for 30 min, and incubated in 100 μl PBS with 0.5% tritonX-100 for 10 min. Then cells were incubated with Apollo solution (Ribobio, Guangzhou, China) for 30 min, followed by DAPI staining for nuclei. EdU-labeled cells were counted manually in ten random fields from each well.

### Immunofluorescence

Cells were fixed with 4% PFA and washed with PBST. Then cells were blocked in PBS with 5% normal goat serum at room temperature for 1h. Samples were then incubated with the primary antibody against Ki67 (No. 14-5698, 1:200, eBioscience, San Diego, USA) at 4°C for overnight. On the next day after washing with PBS, slides were incubated with the secondary antibody (No. A23340, 1:500, Abbkine, USA) at room temperature for 1 h. Afterward, slides were mounted with anti-fade mounting medium (with DAPI) and observed under an epifluorescence microscope.

### Apoptosis analysis

Annexin V-PE /7-AAD apoptosis detection kit (Keygen Biotech, Nanjing, China) was used to analyze cell apoptosis. Cells were washed with ice-cold PBS and stained with annexin-V and 7-AAD. Apoptotic cells were quantified by a dual-color flow cytometry machine (FACS Aria III, BD Biosciences, San Jose, CA, USA). Ten thousand cells were counted per sample. Data were analyzed by Flowjo 7.6 software.

### Cell cycle analysis

The content of DNA was detected by a cell cycle detection kit (Keygen biotech, Nanjing, China). 48 h after lentivirus infection, U251 and U87MG cells were harvested in ice-cold PBS, then fixed with 70% cold ethyl alcohol at 4°C for overnight. Fixed cells were incubated with RNAse A for 30 min and stained with propidium iodide (PI) for 30 min in the darkness. Stained cells were measured by flow cytometry (FACS Aria III, BD Biosciences, San Jose, CA, USA). Data were analyzed with a cell-cycle program (Modfit) to calculate percentages of cells at G0/G1, G2/M and S phases.

### Statistical analysis

All experiments were performed for at least three times independently. All data were presented as mean ± SEM. SPSS 19.0 and Prism 5.0 statistical softwares were employed for the analysis. Corresponding significance levels were indicated in each figure.

## SUPPLEMENTARY MATERIALS FIGURES AND TABLES


