# Supplementary material for: LINC00461, a long non-coding RNA, is important for the proliferation and migration of glioma cells
Source: Oncotarget. 2017 Aug 18;8(48):84123–39. doi: 10.18632/oncotarget.20340 (PMC5663582; doi:10.18632/oncotarget.20340)
Supplement: Supplementary file 1 [file oncotarget-08-84123-s001.pdf]

LINC00461, a long non-coding RNA, is important for the proliferation and migration of glioma cells

SUPPLEMENTARY MATERIALS

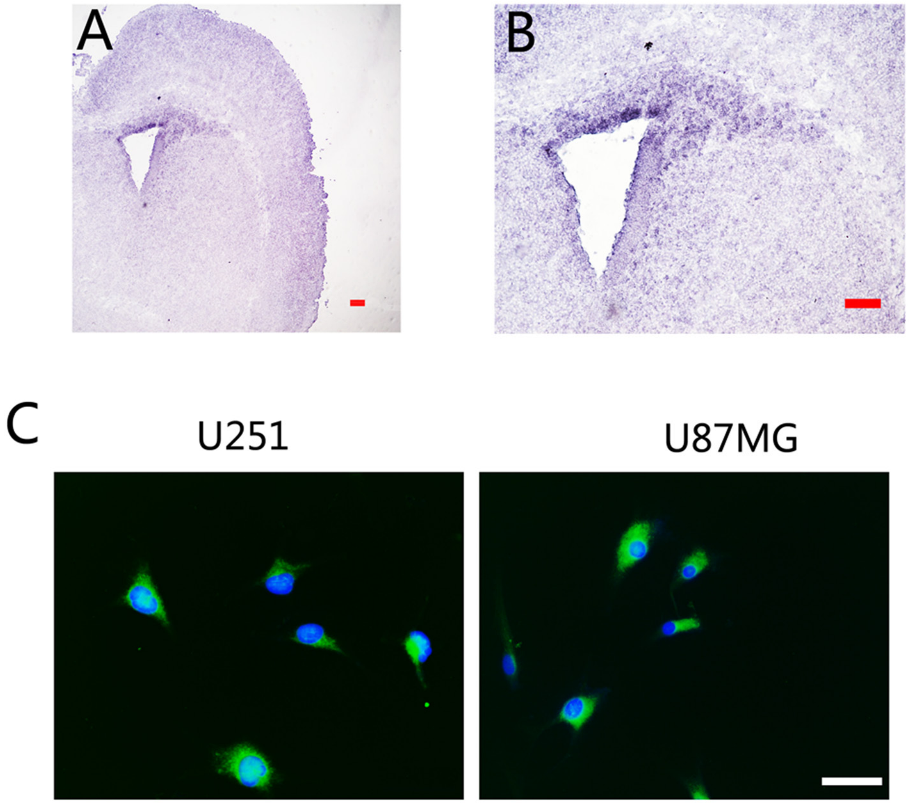

Supplementary Figure 1: The expression of lncRNAs in brain tissues and glioma cells. **(A)** The expression of C130071C03Rik in mouse brain at P0 was detected by *in situ* hybridization. **(B)** is the high power view of A. **(C)** *In situ* hybridization for LINC00461 in U251 and U87 MG cells. Nuclei are counterstained with DAPI. The signal from *in situ* hybridization was presented in artificial green color. Scale bar: 100 μm.

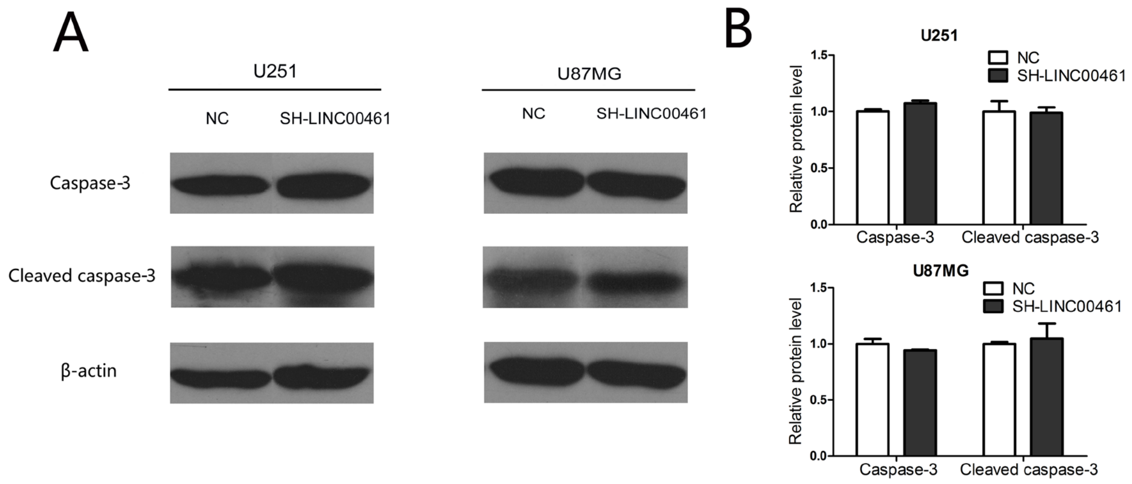

Supplementary Figure 2: The knockdown of LINC00461 had no effects on the expression of caspase-3. **(A)** Expression levels of caspase-3 and cleaved caspase-3 proteins in U251 and U87 MG cells were detected by the western blot assay. β-actin were used as a loading control. **(B)** Histograms showing the quantization of western blot results. Data are presented as mean ± SEM.

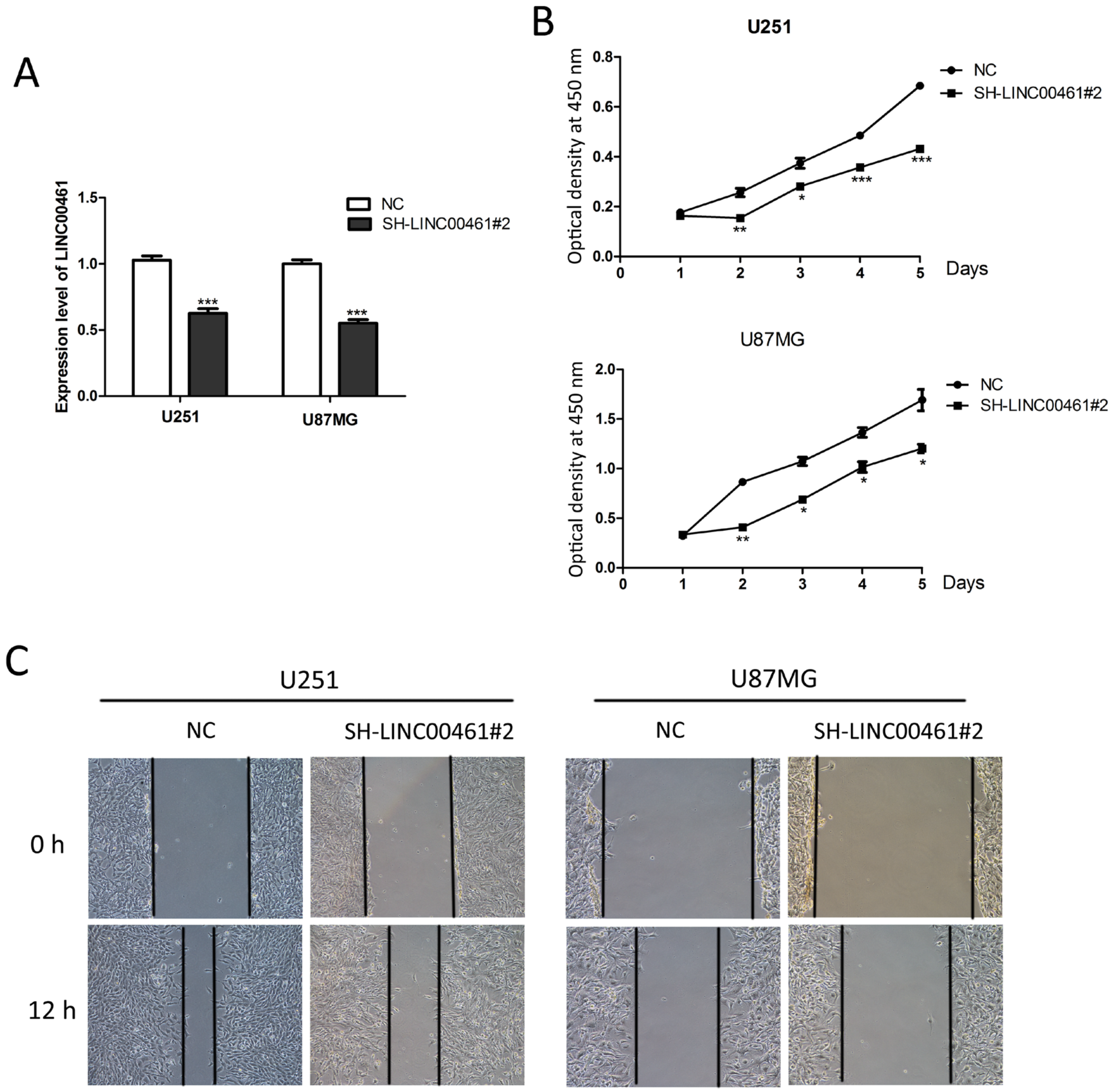

Supplementary Figure 3: LINC00461 knockdown by shRNA#2 inhibited cell viability and migration. **(A)** The efficiency of LINC00461 shRNA#2 was measured by real-time PCR in U251 and U87 MG cells. **(B)** The cell viability was measured by CCK-8 assay. The optical density at 450 nm was used as the positive index of cell viability. **(C)** The wound healing assay showed delayed gap closure in cells with LINC00461 shRNA#2 treatment. Data are presented as mean ± SEM. *, $P < 0.05$; **, $P < 0.01$; ***, $P < 0.001$.

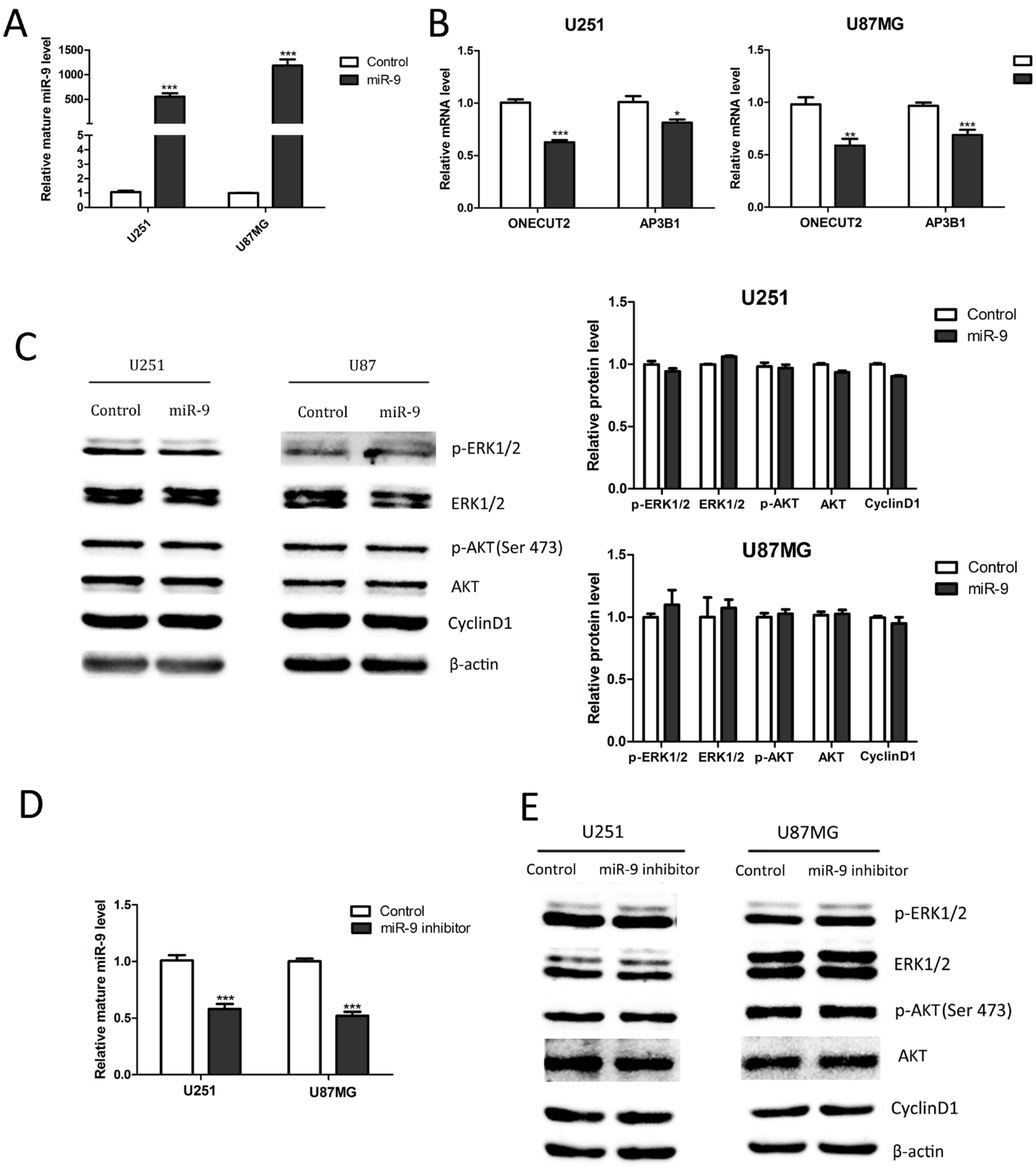

Supplementary Figure 4: Either the overexpression or inhibition of MiR-9 has no effects on ERK and AKT pathways. **(A)** Real-time PCR experiments showed that expression levels of miR-9 increased after miR-9 mimics transfection. **(B)** Real-time PCR experiments showed that expression levels of ONECUT2 and AP3B1 (both are targets of miR-9) decreased after miR-9 mimics transfection. **(C)** Western blot results revealed that expression levels of p-ERK1/2, total-ERK1/2, p-AKT, total AKT and cyclinD1 protein showed no statistic difference in miR-9 overexpression groups compared to those in control groups. β-actin was used as a loading control. **(D)** MiR-9 inhibitor, a 22-bp RNA fragment complementary to miR-9 was applied to inhibited miR-9 expression in glioma cells. A random sequence was used as control. MiR-9 transcripts were significantly suppressed in both U251 and U87 MG cells after miR-9 inhibitor treatment. **(E)** Western blot results revealed that expression levels of p-ERK1/2, total-ERK1/2, p-AKT, total AKT and cyclinD1 protein showed no difference in miR-9 inhibitor groups compared to those in control groups. β-actin were used as a loading control. Data are presented as mean ± SEM. *, P <0.05; **, P < 0.01; ***, P < 0.001.
